# Delivery of IL-12 by neoantigen-reactive T cells promotes antitumor immunity in murine osteosarcoma mode

**DOI:** 10.1093/immadv/ltae010

**Published:** 2024-11-28

**Authors:** Cong Tian, Xingxing Sun, Hongling Zhu, Meixiang Zhou, Qingyu Chen, Daliu Min, Yan Huang, Kun Han

**Affiliations:** Department of Oncology, Shanghai Jiao Tong UniversityAffiliated Sixth People’ s Hospital, Shanghai 201306, China; Department of Oncology, Shanghai Jiao Tong UniversityAffiliated Sixth People’ s Hospital, Shanghai 201306, China; Department of Oncology, Shanghai Jiao Tong UniversityAffiliated Sixth People’ s Hospital, Shanghai 201306, China; Department of Oncology, Shanghai Jiao Tong UniversityAffiliated Sixth People’ s Hospital, Shanghai 201306, China; Department of Oncology, Shanghai Jiao Tong UniversityAffiliated Sixth People’ s Hospital, Shanghai 201306, China; Department of Oncology, Shanghai Jiao Tong UniversityAffiliated Sixth People’ s Hospital, Shanghai 201306, China; Department of Radiation Oncology, Tenth People’s Hospital of Tongji University, Shanghai 200072, China; Department of Oncology, Shanghai Jiao Tong UniversityAffiliated Sixth People’ s Hospital, Shanghai 201306, China

**Keywords:** osteosarcoma, antitumor immunity, IL-12, neoantigen-reactive T cells

## Abstract

**Purpose:**

Despite the proven clinical benefits of cytokine therapy in cancer treatment, systemic administration of cytokines such as IL-12 is constrained by dose-limiting toxicities and short half-lives. To address these challenges, we explored a localized cytokine delivery strategy using engineered neoantigen-reactive T (NRT) cells as carriers in a murine model of osteosarcoma.

**Materials and Methods:**

We used a neoantigen from K7M2 osteosarcoma cells to retrovirally transduce NRT cells to express an inducible form of IL-12. We evaluated the engineered NRT cells’ antitumor activity and the production of IL-12 and IFN-γ upon in vitro co-culture with tumor cells. We systemically administered NRT-IL-12 cells in a mouse model of osteosarcoma to assess their impact on tumor growth and survival.

**Results:**

*In vitro* assays demonstrated that the engineered NRT cells exhibited enhanced antitumor activity and produced elevated levels of IL-12 and IFN-γ. In the mouse model of osteosarcoma, systemic administration of NRT-IL-12 cells resulted in a significant reduction in tumor growth and an increase in survival rates compared to the administration of control NRT cells. Further analysis revealed that NRT-IL-12 cells induced a profound increase in CD8+ T-cell infiltration and a decrease in Treg cells within the tumor microenvironment.

**Conclusion:**

Our study presents a novel and efficacious strategy for osteosarcoma immunotherapy by harnessing NRT cells as targeted cytokine delivery vehicles.

## Graphical Abstract

**Graphical Abstract:**
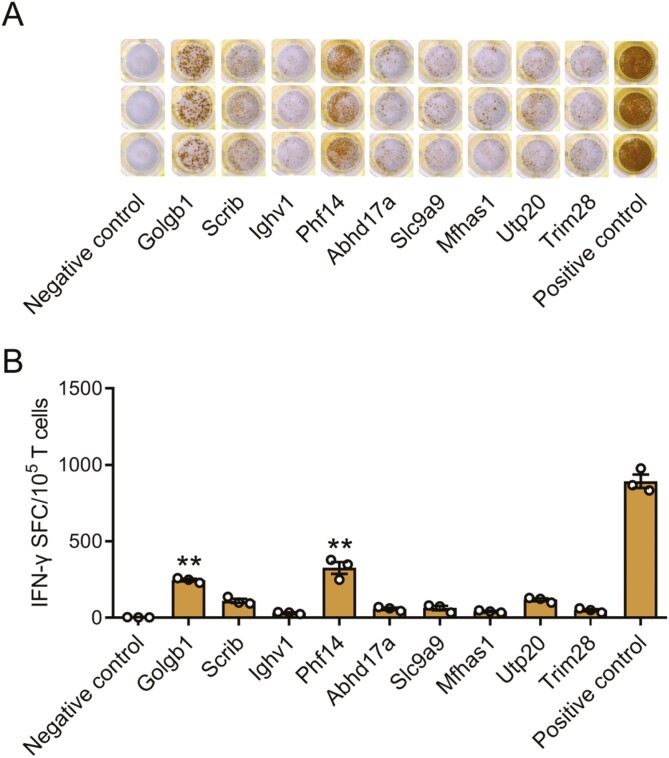

## Introduction

Systemic administration of recombinant cytokines, such as interferon-α (IFN-α) and interleukin-2 (IL-2), is a clinically proven therapeutic strategy for the treatment of cancer [1]. Cytokines perform potent immune modulators by using autocrine and paracrine effects on stromal, cancer, and immune cells within the tumor sites. However, systemic administration of cytokines has faced several limitations in the clinic. Due to their short half-lives, frequent administration is required in clinical applications, which often results in severe toxicity [2]. These limitations may be overcome through local delivery to increase the concentration of cytokines in the tumor sites, promote an antitumor immune response, and reduce systemic toxicity. Local cytokine administration has been evaluated using viral and nonviral gene therapy vectors [3, 4]. However, intratumoral injection is only applicable for tumors with accessible lesions, which can be challenging for many tumor types.

Using cells as drug carriers holds great promise for antitumor therapy. Some advantages of using cells as drug carriers include enhancing targeting, enhancing the penetrability of drugs to tumor cells, and reducing systemic toxicities [5, 6]. Introducing cytokines into tumors via engineered immune cells, such as CAR-T, TCR-T, or tumor-infiltrating lymphocytes (TILs) is a very promising antitumor approach [7, 8]. However, using these T cells as cytokine carriers can still present challenges, such as many solid tumors do not have suitable CAR-T targets, the preparation of TCR-T is complicated and expensive, and TILs can have off-target toxicity [9]. Neoantigens are antigens produced by tumors due to genetic mutations, making them exquisitely tumor-selective. Neoantigen-reactive T cells (NRT) are therefore ideal cell carriers for cytokines [10].

IL-12 is a proinflammatory cytokine that bridges innate and adaptive immunity [11]. Preclinical studies in mouse tumor models have confirmed the antitumor activity of IL-12, which can induce tumor regression or prolong the survival time of tumor-bearing animals [12]. However, systemic use of IL-12 in clinical trials resulted in serious unexpected side effects, which greatly limits its clinical application [13]. Gene therapy is one option for expressing IL-12 locally in tumors. Both viral and nonviral IL-12 vectors have demonstrated high antitumor efficacy in mouse tumor models. Some IL-12 vectors had shown low toxicity in clinical trials, but only moderate antitumor effects had been achieved [14].

In this study, using a murine osteosarcoma model with a low mutation load, NRT cells were successfully prepared and transduced with an inducible IL-12 retrovirus expression vector. *In vitro* and *in vivo* tests showed that expression of IL-12 by NRT cells had stronger anti-tumor effects compared to control NRT cells.

## Materials and methods

### Cell lines and mice

Six- to eight-week-old sex-matched BALB/C mice were purchased from Hangzhou Ziyuan Experimental Animal Technology Co., Ltd. All animal experiments were performed under specific pathogen-free conditions. The animal experiments were approved by the Animal Experimental Ethics Committee of Shanghai Jiao Tong University Affiliated Sixth People’ s Hospital, Shanghai, China.

K7M2 and F420 cell lines were obtained from the American Type Culture Collection. The cells were cultured in Dulbecco’s Modified Eagle Medium supplemented with 15% fetal bovine serum (FBS), 100 U/ml penicillin, and 100 µg/ml streptomycin at 37°C and in the presence of 5% CO_2_.

### Neoantigen prediction

Previously published exome (PRJNA698961)and transcriptome (GSE166282) data of K7M2 cells were downloaded from the National Center for Biotechnology Information (NCBI) database [15]. Neoantigen was predicted using the MuPeXIpipeline [16]. Each peptide was provided a score on the basis of HLA-binding affinity, expression level, similarity to self-peptides, and mutant allele frequency. Peptides with a priority score of > 0 were selected as neoantigen candidates.

### Synthesis of long peptides and final vaccine preparation

Peptides with purity greater than 95% were synthesized by GL Biochem (Shanghai, China) using fluorenyl methoxy carbonyl chemistry by reverse-phase high-performance liquid chromatography and were confirmed by mass spectrometry. The lyophilized peptides were dissolved in dimethyl sulfoxide, diluted in phosphate-buffered saline (pH 7.4) to a concentration of 10 mM, and stored as aliquots at −80°C as described previously.

### Generation of neoantigen-reactive T cells

The preparation of bone marrow-derived DCs cells and CD3^+^T cells from K7M2 tumor-bearing BALB/C mice were performed as described previously [17]. For NRT populations containing mutation-reactive T cells, we attempted to isolate those T cells by fluorescence-activated cell sorting (FACS) CD3^+^CD137^+^ cells after stimulation with autologous peptide-pulsed DCs. Peptide-pulsed DCs were incubated overnight with T cells in 24-well plate wells. The cocultures were then stained with anti-CD3 and anti-CD137, and cells were washed once prior to acquisition. CD3^+^CD137^+^ cells were sorted using BD FACSAria.

Sorted T cells were expanded by restimulating with peptide-pulsed DCs at an interval of 7 days in a medium containing 30 ng/ml anti-CD3 (OKT3) and 3000 IU/ml IL-2. Half of the medium volume was replaced every 3 days with fresh medium containing IL-2 (3000 IU/ml). After the third stimulation, NRT cells were harvested and re-suspended with phosphate-buffered saline (PBS).

### Construction of inducible IL-12 vectors

To generate inducible murine IL-12 vectors, named mNFAT-IL-12, which consisted of six repeats of murine NFAT-binding motif and a murine single-chain IL-12 as well as a PA2 in tandem. The murine single-chain IL-12 was generated by fusing the murine IL-12p40 and murine IL-12p35 subunits with a (Gly4Ser)3 linker [18].

### Retrovirus production

For retrovirus production, the 293T cells were seeded at 1.5 × 10^7^ per 15-cm dish. On the next day, 293T cells were transfected with the different recombinant murine expression vectors encoding mNFAT-IL-12 together with the retroviral packaging plasmid pCL-Eco (Addgene) by using a polyethyleneimine-based DNA transfection reagent. Also, the medium was changed 6 h later. The viral supernatants were harvested at 48 h after transfection [18].

### Transduction of T cells

NRT cells were activated with 50 ng/ml OKT3 (Ortho Biotech, Horsham, PA) for 2 days and transduced with retrovirus as described [18]. Brieﬂy, cells were harvested on day 2 poststimulation and then transduced with retroviruses on RetroNectin-coated 24-well plates. After transduction, the cells were cultured in RPMI1640 medium supplemented with 10% FBS and IL-2 (300 U/ml) until use.

### ELISPOT assay

ELISPOT kits (BD Biosciences) were used to determine the amount of cytokine-secreting T cells after overnight activation with a peptide [19]. In this study, a multiple culture protocol was used to analyze the T cell response as described above. Briefly, the DC-pulsed peptide coculture with T cells (10^5^ per well) was added to wells for 18–20 h. The plates were washed before the addition of the diluted detection antibody (1:100 dilution) and then incubated for 1 hour at 37°C. After the plates were washed, streptavidin-HRP (1:100 dilution) was added, and the plates were incubated at 37°C for 1 h. 3-Amino-9-ethylcarbazole (AEC) solution mix was then added to each well, and the plates were left in the dark for approximately 15–25 min at room temperature. Deionized water was then added to stop the reaction, and the plates were subsequently scanned using an ELISPOT CTL Reader (Cellular Technology Inc.). The results were analyzed with ELISPOT software (AID). Spots with a size that were more than twofold greater than that of the no-peptide control (medium only) were considered positive for T-cell reactivity.

### Flow cytometry

The anti-mouse monoclonal antibodies used for cell surface staining were CD3-APC and CD137-PE (clone: 41BB). All the antibodies were obtained from BioLegend (San Diego, CA). Briefly, NRT cells were co-cultured with the K7M2 cells in a ratio of 2:1. After 8 h, NRT cells were collected, centrifuged at 300 g for 10 min, and the cell pellet was washed with FACS buffer, and then incubated for 30 min with the antibodies on ice, washed in FACS buffer, and read as the relative log ﬂuorescence of live cells using MACSQuant ﬂow cytometer (Miltenyi Biotech). Samples were analyzed using FlowJosofware (BD Bioscience).

### Cytokine release assays

NRT cells were cocultured with tumor cells in different ratios. Twenty-four hours later, supernatant was collected. The levels of murine IFN-γ, TNF-α, and IL-12 secreted by NRT cells were detected by using a commercially available ELISA kit (MultiSciences Biotech) according to the manufacturer’s manual. For serum cytokine release assay, mouse sera and tumor tissues were collected and measured by ELISA kits.

### Cytotoxicity assays

Different target cancer cells were cocultured with the NRT cells at effector/target ratios of 1:1, 5:1, and 10:1. Following 24 h of coculture, the levels of supernatant lactate dehydrogenase were tested by the CytoTox 96 Non-Radioactive Cytotoxicity Kit (Promega) in accordance with the manufacturer’s instructions.

### Adoptive cell transfer in a murine model

BALB/C mice at 6 to 12 weeks of age were injected with 5 × 10^5^ K7M2 cells. Seven days later, groups of tumor-bearing mice were transferred with NRT cells by tail vein injection. The tumor size and body weight of the mice were observed every 2–3 days. The mice were killed when the tumor volume reached 1500 mm^3^. The NCI Animal Care and Use Committee of the National Institutes of Health approved all animal experiments.

For the IL-12 neutralizing experiment, the neutralizing antibody (clone C17.8, BioXCell) was intraperitoneally injected at a dose (38.8 mg/kg body weight) one day before therapy.

### Immunohistochemistry assays

All samples for immunohistochemistry (IHC) assays were fixed with formalin and embedded in paraffin. Next, serial sections were performed. To detect mouse CD8^+^T cells and Treg cells, the sections of formalin-fixed, paraffin-embedded tumor tissue were immunostained with anti-mouse CD8 and Foxp3 mAb (eBioscience) followed by peroxidase-conjugated secondary Abs (ChemMate Dako Envision Detection Kit, Peroxidase/DAB, Rabbit/Mouse; Dako). Briefly, after deparaffinization and blocking of endogenous peroxidase, Ag retrieval was achieved by microwave using sodium citrate solution (pH 6). Subsequently, the slices were blocked in BSA (1%) for 30 min at room temperature and immunostained with anti-mouse mAb at 1:500 dilution overnight at 4°C. Next, the sections were washed with PBS and incubated with goat anti-rat IgG-HRP (Santa Cruz Biotechnology) for 1 h and counterstained with hematoxylin.

### Statistical analysis

The two-tailed Student’s *t*-test was used to determine the statistical significance of the differences between means. All statistical tests were performed using SPSS manager software. GraphPad Prism 5.0 was used for statistical calculations, and *P* < .05 (*), *P* < .01 (**), and *P* < .001 (***) were considered significant.

## Results

### Immunogenic neoantigens were predicted and identified from K7M2 cells

**Figure 1.**
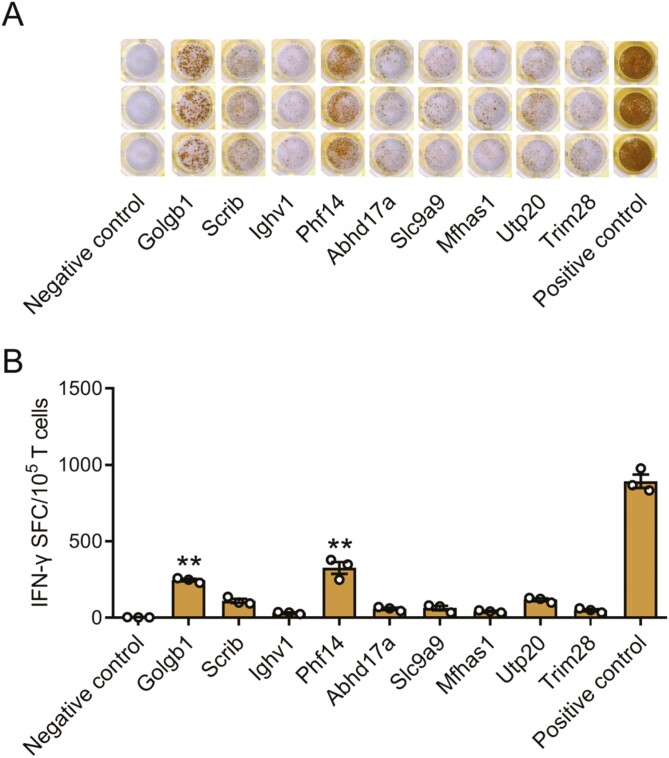
Immunogenicity testing of neoantigens from K7M2 cells (**A, B**) Splenocytes from K7M2 cells inoculated mice were stimulated with the peptide-pulsed DCs for 18-20 h.T-cell responses to each antigen were measured by an IFN-γ ELISPOT assay. Data are presented as the mean ± S.D. of three independent experiments. **P < .01 compared with IFN-γ production by splenocytes stimulated without peptide. SFC, spot-forming cell.

We performed neoantigen analysis of the mutations encoded by K7M2 cells using theMuPeXI pipeline. Ten mutation-associated long peptides (27 amino acids, centered on the mutation in position 14) were selected according to the comprehensive score and produced by chemosynthesis (9 neoantigens were synthesized successfully, Table 1). To identify the neoantigen with the highest immunogenicity, we performed an ELISPOT assay using bone-marrow-derived dendritic cells pulsed with each of the synthesized mutation-associated long peptides. T cells were isolated from spleens of K7M2 tumor-bearing mice. The neoantigens Golgb1 and Phf14 elicited the most significant peptide-specific T-cell responses and were thus selected for further studies (Fig. 1A, B).

**Table 1.** Neoantigens identified from K7M2 cells.

Gene symbol	Amino acid change	HLA allele	Augmented peptide	Priority score
Golgb1	K/N	H-2-Kb	TLMEKVELEVAER**N**LSFHNLQEEMHQL	72.7
Scrib	N/T	H-2-Db	NDVSLQALPGDVG**T**LANLVTLELRENL	71.6
Ighv1	F/L	H-2-Kb	INPSSGYTKYNQK**L**KDKATLTADKSSS	52.1
Phf14	C/F	H-2-Kb	AAEEDIADPFFAY**F**KQHADRLDRKWKR	50.9
Abhd17a	G/A	H-2-IAb	SLVPEPEPGPGGA**A**AAPSGPLRTSAAT	44.3
Slc9a9	F/I	H-2-Kb	AENVIFCYMGLAL**I**TFQNHIFNALFIL	43.7
Slc4a1ap^a^	G/R	H-2-Db	EQGFYLYDLGSTH**R**TFLNKTRIPPRTY	43.5
Mfhas1	R/G	H-2-IAb	PTTVGSFLHRVGA**G**VPHAVVCIVGTHA	42.4
Utp20	L/V	H-2-Kb	HLISTYLPKILQI**V**LCMTATVSHILDQ	41.9
Trim28	P/L	H-2-IAb	AAAGQAGTVPPGA**L**GAPPLPGMAIVKE	36.8

^a^Not synthesized successfully.

### Preparation of neoantigen-reactive T cells (NRT)

**Figure 2.**
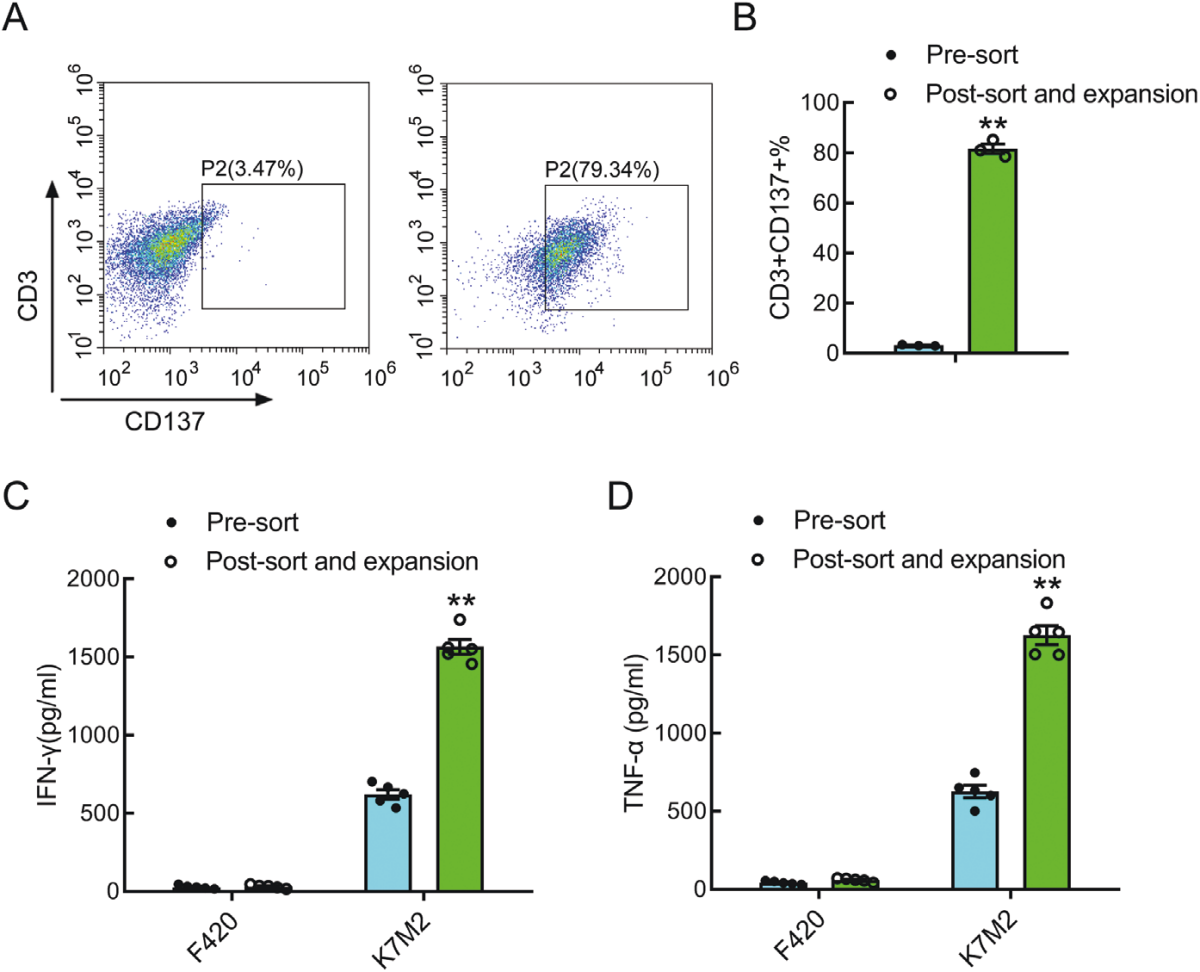
Preparation of Golgb1 and Phf14 peptide-induced NRT cells (**A, B**) NRT cells were sorted and co-cultured with Golgb1 and Phf14 peptide-pulsed DCs for 28 days. NRT cells were co-cultured with K7M2 cells for 8 h. Activated T-cell percentage (CD3^+^/CD137^+^T-cell population) in NRT cells were measured by flow cytometry. (**C, D**) NRT cells were co-cultured with K7M2 or F420 cells in a 96-well plate overnight. Cells were centrifuged, supernatant was collected, and the secreted IFN-γ and TNF-α levels were determined through ELISA. **P < .01.

To produce NRT cells elicited by Golgb1 and Phf14, T cells were obtained from spleens of K7M2 tumor-bearing mice and stimulated with Golgb1 and Phf14 peptide-pulsed DCs. CD137, also known as 4-1BB, is a co-stimulatory receptor expressed on recently activated T cells [20]. We used CD137 as a marker to sort antigen-specific T cells by FACS. Sorted T cells were then expanded by restimulating with peptide-pulsed DCs every 7 days in a medium containing anti-CD3 and IL-2. Flow cytometry analysis showed that the percentage of CD137^+^ T cells increased from 3.2% in input spleen T cells to 81.6% i after three rounds of stimulation with Golgb1 and Phf14 peptide-pulsed DCs (Fig. 2A, B).

The ability of NRT cells to mediate effector functions in response to K7M2 cells *in vitro* was evaluated through IFNγ and TNFα production as determined by ELISA of culture supernatants. As shown in Fig. 2C–D, NRT cells produced significantly moreIFNγ and TNFα upon co-cultured with K7M2 cells as compared to control F420 cells that lack expression of the Golgb1 and Phf14 neoantigens. Thus, our experiments confirmed that our expansion protocol efficiently generates K7M2-specific NRT cells.

### Generation of neoantigen-reactive T cells secreting inducible IL-12

**Figure 3.**
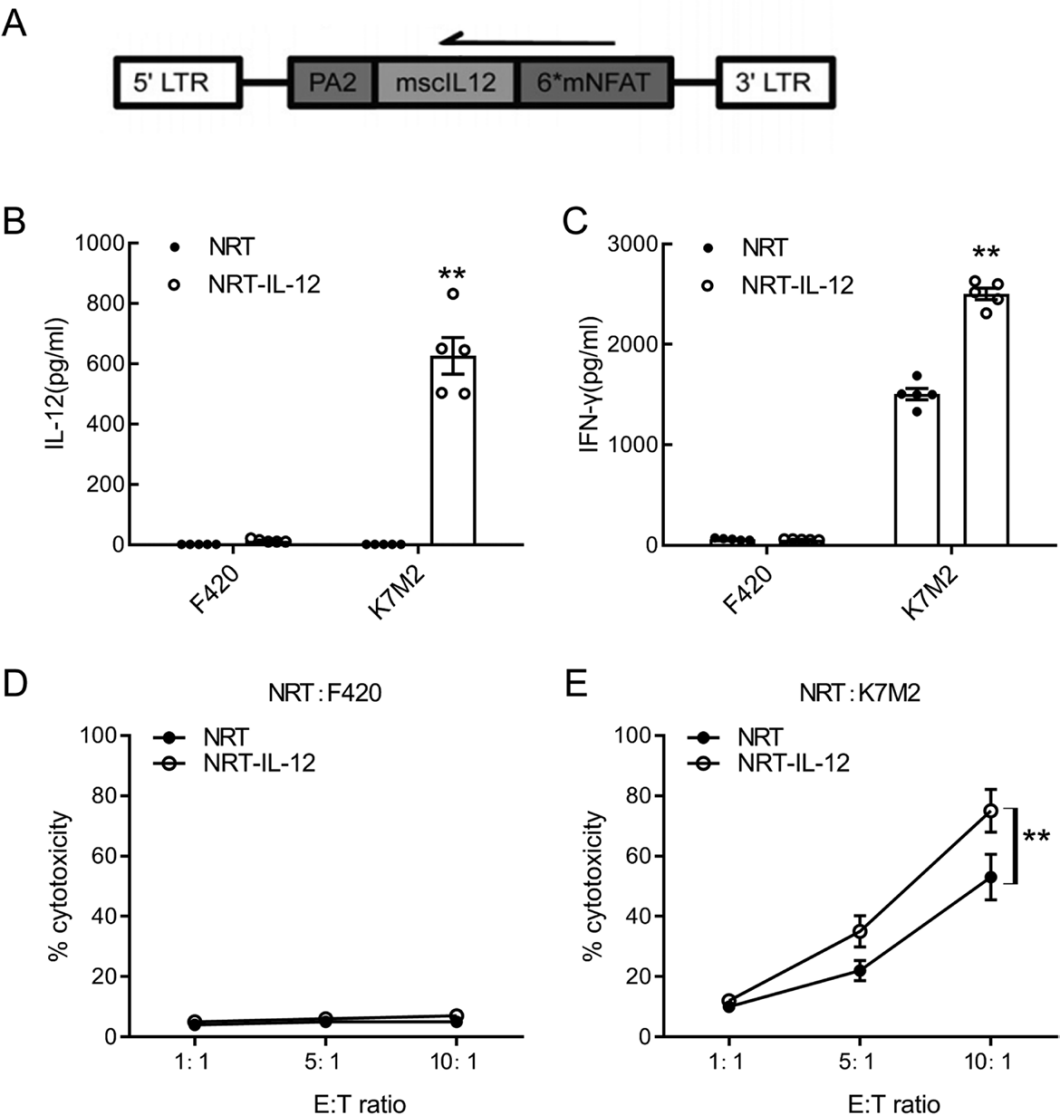
Activation-dependent IL-12 production and *in vitro* function of NRT- IL-12 cells. **(A)** Schematic representation of retroviral vectors expresses IL-12 under the transcriptional control of the inducible promoter that contains 6 mNFAT-binding motifs. (**B**,**C**) The secretion of IL-12 and IFN-**γ** of NRT cells following 24 h coculture with K7M2 or F420 cells at 1:1 ratio was determined by ELISA. (**D**) *In vitro* cytotoxicity of NRT cells incubated with F420 cells at 1:1, 5:1, and 10:1ratios for 18 h. (**E**) *In vitro* cytotoxicity of NRT cells incubated with K7M2 cells at 1:1, 5:1, and 10:1 ratios for 18 h. Data are presented as the mean ± S.D. of five independent experiments. **P < .01.

To mitigate the toxicity caused by the constitutive expression of IL-12, we constructed an inducible promoter for IL-12 that had a low basal activity but could be activated by TCR engagement. The schematic representation of the retroviral vector constructs used in this study is shown in Fig. 3A. IL-12 expression is controlled by an NFAT-responsive promoter that is activated in response to TCR signaling [18]. In subsequent experiments. NRT-IL-12 cells were compared to control untransduced NRT cells.

To test if IL-12 secretion could be driven by T-cell activation, NRT cells were stimulated with K7M2 cells expressing the target neoantigens or F420 cells as a negative control. Activated NRT-IL-12 cells expressed significantly more IL-12 than untransduced NRT cells when cocultured with K7M2 cells but not F420 cells (Fig. 3B). Meanwhile, we assessed the production of other cytokines by NRT cells. Secretion of IFN-γ was significantly higher in NRT-IL-12 cells compared with untransduced NRT cells when cocultured with K7M2 cells but not F420 cells (Fig. 3C). Cytotoxicity assays revealed that NRT cells could kill K7M2 cells but not F420 cells (Fig. 3D). Moreover, NRT-IL-12 cells could kill K7M2 cells more effectively thanuntransduced NRT cells (Fig. 3E), suggesting that IL-12 production enhances multiple CD8 T-cell effector functions.

To verify whether these increased effector functions were IL-12-dependent, IL-12was neutralized with an anIL-12 antibody, and we found that the increased expression of IFNγand the increased cytotoxicity of NRT-IL-12 cells when exposed to K7M2 cells disappeared after IL-12 neutralization (Supplementary Fig. S1A, B).

### Neoantigen-reactive T-IL-12 cells exhibits antitumor therapy in the mouse osteosarcoma model in IL-12-dependent manner

**Figure 4.**
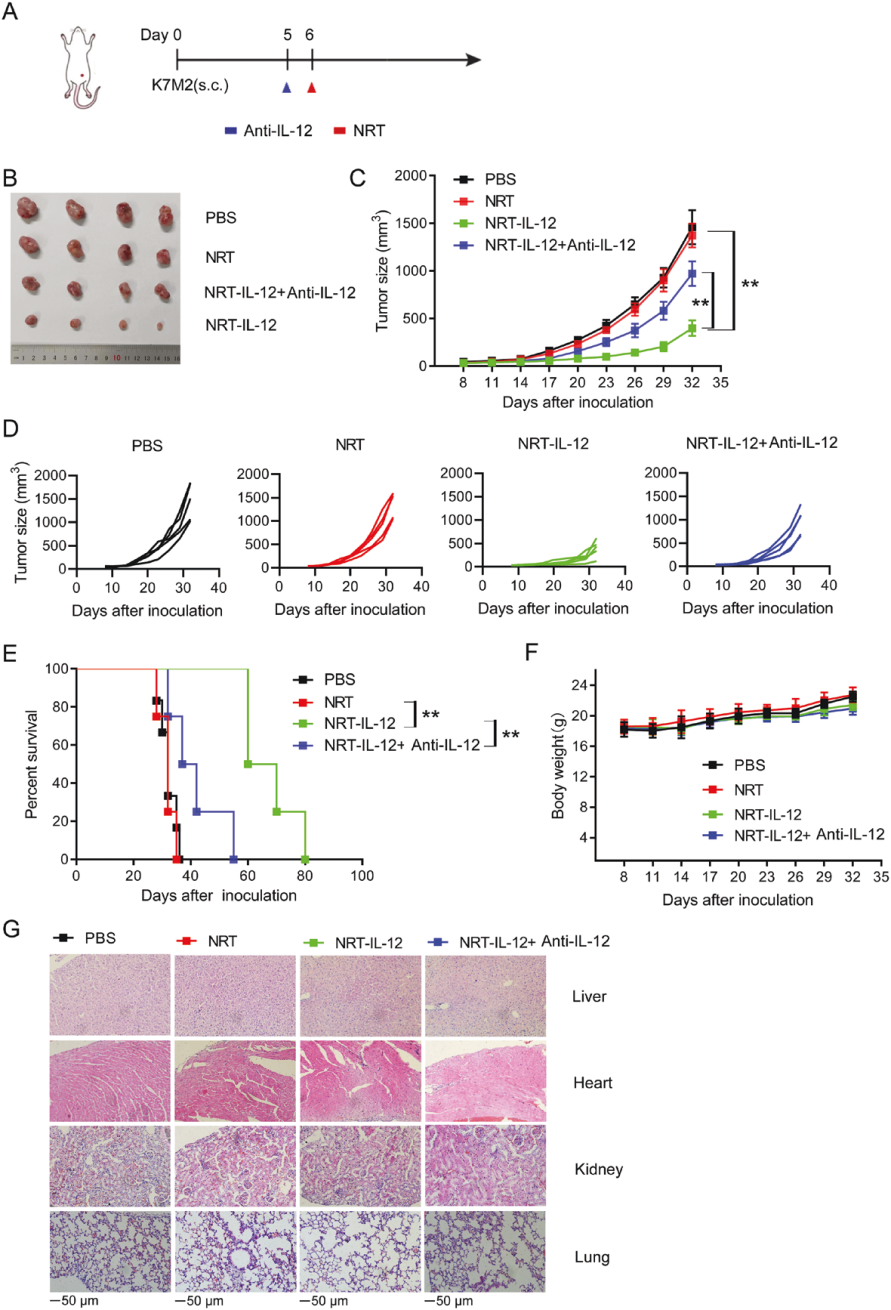
NRT-IL-12 cells improved antitumor immunity against established murine K7M2 tumor by an IL-12-dependent manner. (**A**) Experimental scheme of the in vivo antitumor experiment. On day 0, 5 × 10^5^K7M2 cells were subcutaneously injected into 6-week-old female BALB/C mice. On day 8, mice were infused intravenously with 1 × 10^7^NRT cells, For IL-12 neutralizing, the neutralizing antibody was intraperitoneally injected at a dose (38.8 mg/kg body weight) one day before therapy. (**B, C, D**) tumor growth was observed every 3 days and recorded as the mean tumor size (mm^3^). (**E**) Kaplan–Meier survival curves of mice treated as indicated. (**F**) Body weight of each group was measured every 3 days. (**G**) Representative images of histopathological evaluation of major organs using H&E staining analysis: the tissue structures of the heart, lungs, liver, and kidneys, removed from the mice on day 17, remained normal in all groups. **P < .01.

To explore the antitumor efficacy of NRT-IL-12 cells *in vivo*, BALB/C mice were inoculated subcutaneously with K7M2 cells. NRT-IL-12 cells were administered intravenously without prior conditioning when tumors grew to 100 mm^3^ (Fig. 4A). As shown in Fig. 4B–D, although NRT cells could kill tumor cells *in vitro*, they had no such effect *in vivo*. However, tumors in mice treated with NRT-IL-12 cells grew more slowly than those in mice treated with control untransduced NRT cells. Neither NRT nor NRT-IL-12 cells were effective against F420 cells *in vitro* (Fig. 3D), and they were similarly ineffective *in vivo* (Supplementary Fig. S2).

In order to verify whether the observed tumor control is IL-12-dependent, IL-12 expression in mice was neutralized by IL-12 antibody, and we found that the antitumor effect of NRT-IL-12 cells disappeared after IL-12neutralization(Fig. 4B–D). In addition, tumor-bearing mice treated with NRT-IL-12 cells had prolonged survival when compared with control groups (*P* < 0.05, Fig. 4E).

Systemic administration of IL-12 is toxic in both mice and humans. In our system where IL-12 expression is restricted to activated tumor-specific T cells, body weight of mice in all groups increased over time, and there were no statistically significant differences among groups (Fig. 4F). Histopathological analysis of major organs (heart, lungs, kidneys, and liver) using H&E staining showed that the tissue structures in all groups of mice remained normal, although some organs showed mild chronic granulocyte infiltration but no significant damage(Fig. 4G). There were no statistically significant differences between groups in major biochemical indicators such as AST, ALT, and UREA (Supplementary Fig. S3). These findings suggest that the use of NRT-IL-12 therapy would be less likely to induce immunologically related adverse events, although this would require testing in humans.

### Delivery of IL-12 by neoantigen-reactive T cells into tumors promotes CD8^+^ T cell infiltration and a decrease in Tregs

**Figure 5.**
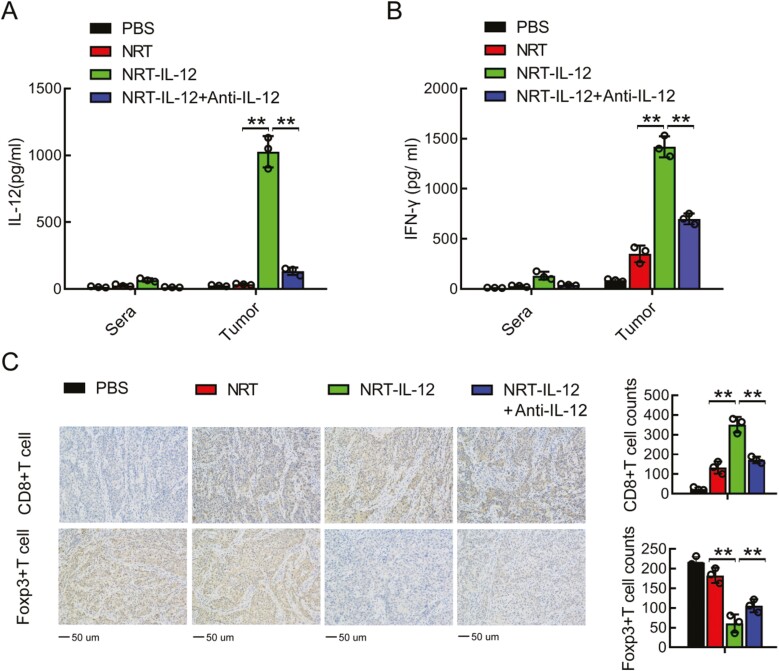
Inducible IL-12 enhanced NRT cell infiltration and decreased Treg infiltration. (**A, B**) The amounts of IFN-γ and IL-12 in the sera and tumors of treated mice 8 days after therapy were assessed by ELISA. (**C**) Infiltration of CD8+T and Treg cells in tumors 8 days after therapy was measured by immunohistochemistry assay. The data shown are from three independent experiments. **P < .01.

To identify the mechanisms accounting for the pronounced antitumor efficacy of NRT-IL-12 cells, we measuredIFNγ and IL-12 in sera and tumor. IL-12 was elevated in tumors of mice treated with NRT-IL-12 whereas it was barely detectable in serum and tumor of mice treated with PBS or control untransduced NRT cells (Fig. 5A). IFNγ was significantly elevated in tumors of mice treated with NRT-IL-12 compared to untransduced NRT cells, and IFNγ was barely detectable in serum of all mice (Fig. 5B). As expected, elevated levels of IL-12 and IFNγ induced by NRT-IL-12 cells disappeared upon neutralization of IL-12 (Fig. 5A, B). Our results showed that the concentrations of IL-12 and IFN-γ in tumors were much higher than those in blood, consistent with our approach to local production of IL-12 in the tumor microenvironment by activated T cells as a means of avoiding systemic exposure.

Previous studies found that increased intratumoral IL-12 could promote CD8^+^ T-cell infiltration and decrease infiltration of Treg cells, thereby extending survival [21, 22]. In our model, we assessed the abundance of CD8^+^ T cells and Tregs in tumor tissue 8 days after infusion of NRT cells. Compared with tumors treated with PBS or control untransduced NRT cells, tumors in mice receiving NRT-IL-12 cells were infiltrated with more CD8^+^ T cells and fewer Foxp3^+^ cells (Fig. 5C). In addition, increased CD8^+^ T-cell infiltration and decreased Treg infiltration in tumor were diminished by IL-12 neutralization (Fig. 5C).

Collectively, our results demonstrated that NRT-IL-12 cells could be induced to secrete IL-12 in tumors, leading to augmented IFNγ secretion, increased CD8^+^ T-cell infiltration, decreased Treg infiltration, and further augmentation of antitumor immunity.

## Discussion

In this study, mouse osteosarcoma K7M2 cells were analyzed and several highly immunogenic neoantigens (Golgb1 and Phf14) were identified. We used Golgb1 and Phf14 peptide-pulsed DCs to expand neoantigen-reactive T cells and also transduced them to conditionally express IL-12 upon activation. NRT-IL12 cells showed superior tumor control both *in vitro* and *in vivo*, suggesting that this approach has translational potential.

Osteosarcoma is the most common bone cancer in young adults, adolescents, and children. Using the current standard of care surgery and chemotherapy, about 70% of patients with newly diagnosed and localized diseases can achieve long-term remission [23]. Unfortunately, patients with metastatic or relapsed disease have overall survival rates <20% despite being treated with multimodal therapy [24]. Immune checkpoint inhibitors (ICIs) have thus far failed to improve outcomes for osteosarcoma patients [25]. Effective and safe treatments for osteosarcoma patients are urgently needed. Cytokines, such as GM-CSF [26], IL-2 [27], and IFN-α [28], are clinically validated therapeutic strategies for the treatment of osteosarcoma. However, systemic administration of these cytokines is accompanied by severe toxicities, including hypotension, oliguria, edema, and hepatitis which restricts the use of these therapies [29]. Local delivery of cytokines to the tumor to exert antitumor effects poses an attractive alternative. Here, we showed that expression of IL-12 enhanced tumor killing activity of neoantigen reactive T cells *in vivo*. Importantly, the expression of IL-12 was restricted to the tumor site, thus obtaining a strong antitumor effect, and a favorable safety profile.

IL-12 is an inflammatory cytokine that is produced by DCs, macrophages, and neutrophils [30]. IL-12 is one of the most robust antitumor cytokines in various murine tumor models [13]. However, systemic administration of IL-12 leads to severe toxicities, including deaths, which extremely limited its clinical application [14, 31]. Intra-tumoural injection of IL-12 expressing vectors, IL-12 mRNA transiently engineered T cells in multiple preclinical and clinical studies demonstrate antitumor efficacy without any significant toxicity [32–36]. However, intratumor injection is challenging for tumors such as osteosarcoma that are not easily accessible. Several preclinical and clinical studies have demonstrated the enhanced antitumor activity of T cells, which secreted IL-12 constitutively or inducibly [18, 21, 37–39]. Given that TILs are comprised of both tumor-specific and bystander T cells, IL-12 delivery by total TIL cells has the potential for off-target reactivity leading to unwanted side effects [38]. Our study demonstrated that IL-12 delivery by neoantigen-reactive T cells could significantly enhance the expression of IL-12 in tumor sites and promote the antitumor effect of neoantigen-reactive T cells in osteosarcoma without significant toxicity.

Neoantigens, which are derived from somatic mutations and recognized by T cells as foreign, are attractive targets for adoptive T-cell therapy. They are expressed exclusively in cancer cells, making them truly tumor-specific [40]. Neoantigen-reactive T cells have been detected in tumor tissues and blood of patients [41, 42]. However, the effectiveness of adoptive neoantigen-reactive T therapy in osteosarcoma has not been reported. Our results show that osteosarcoma-specific NRT cells have a strong antitumor effect in vitro. However, the therapeutic effect of osteosarcoma-specific NRT cells is still very limited in mouse models, potentially due to the immunosuppressive effects of the tumor microenvironment [43]. Other possible reasons include a lack of lymphodepletion chemotherapy before NRT cell infusion and a lack of IL-2 support for the adoptively transferred T cells, as performed in other studies [44]. Nevertheless, neoantigen reactive T cells could serve as delivery vehicles for IL-12, thus augmenting IFN-γ secretion, increasing CD8^+^T-cell infiltration, decreasing Treg infiltration, and further promoting antitumor immunity. Neoantigen reactive adoptive Tcell therapy has been used in many clinical trials [10], but the clinical effect has been limited. Our approach provides a novel and efficacious strategy for tumor immunotherapy.

Our study also has some limitations. We did not investigate the number of NRT cells for adoptive transfer therapy. Further research is needed to explore the appropriate dose range required to achieve the desired therapeutic effect without causing toxicity. In addition, our study was limited to mouse osteosarcoma models. Whether similar results would be observed in other tumor types remains to be studied.

Together, we successfully expanded neoantigen reactive T cells for osteosarcoma, a tumor with a relatively low mutational burden, and we used these neoantigen reactive T-cells as carriers for local delivery of IL-12. We believe that this approach presents a novel and efficacious strategy for tumor immunotherapy by harnessing neoantigen reactive T cells as targeted cytokine delivery vehicles.

## Supplementary Material

ltae010_suppl_Supplementary_Figures_S1

ltae010_suppl_Supplementary_Figures_S2

ltae010_suppl_Supplementary_Figures_S3

## Data Availability

The data generated in this study are available within the article.
