# Supplementary figures and images for: Delivery of IL-12 by neoantigen-reactive T cells promotes antitumor immunity in murine osteosarcoma mode
Source: Immunother Adv. 2024 Nov 28;5(1):ltae010. doi: 10.1093/immadv/ltae010 (PMC11684073; doi:10.1093/immadv/ltae010)

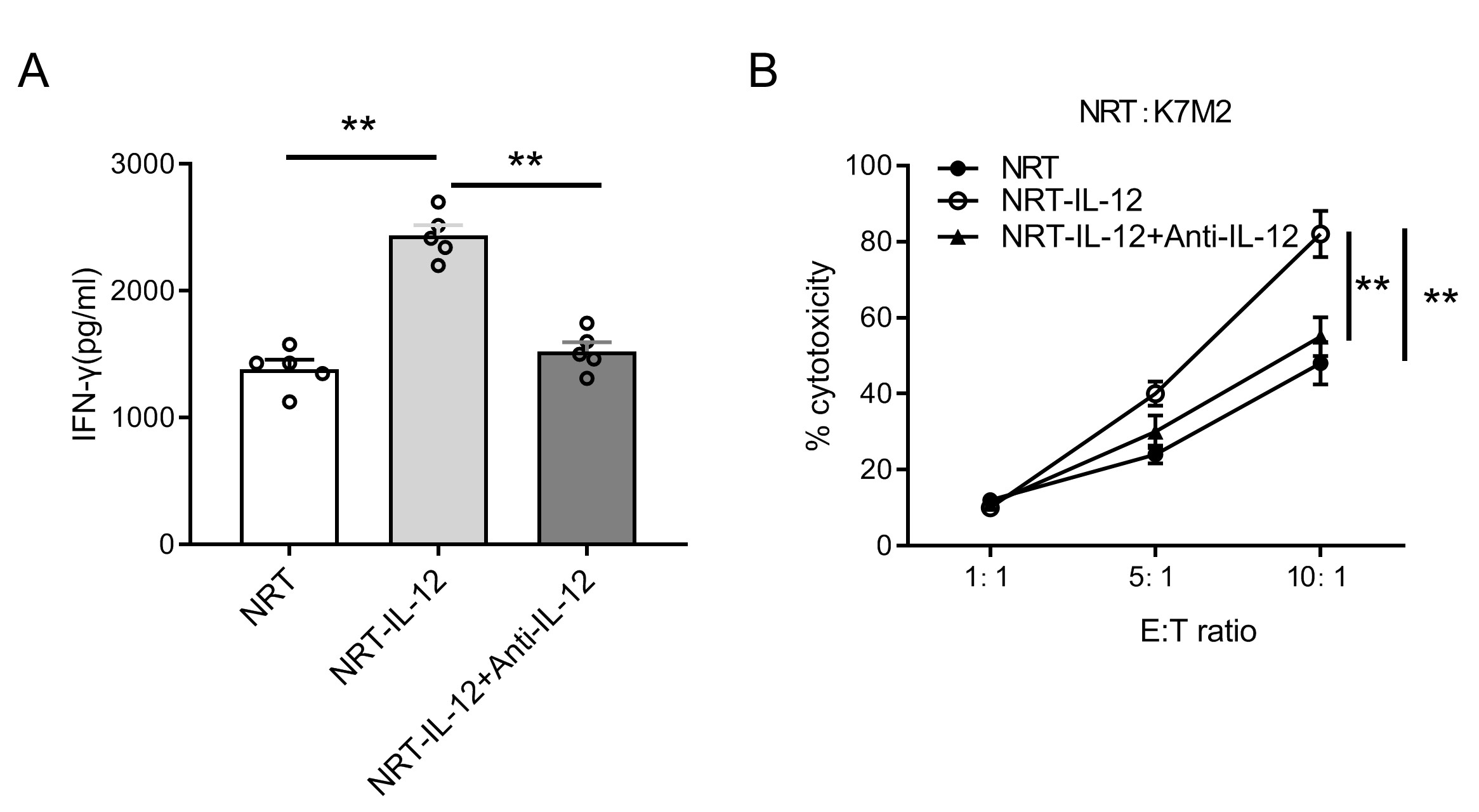

Supplement: ltae010_suppl_Supplementary_Figures_S1 [file ltae010_suppl_supplementary_figures_s1.jpeg]

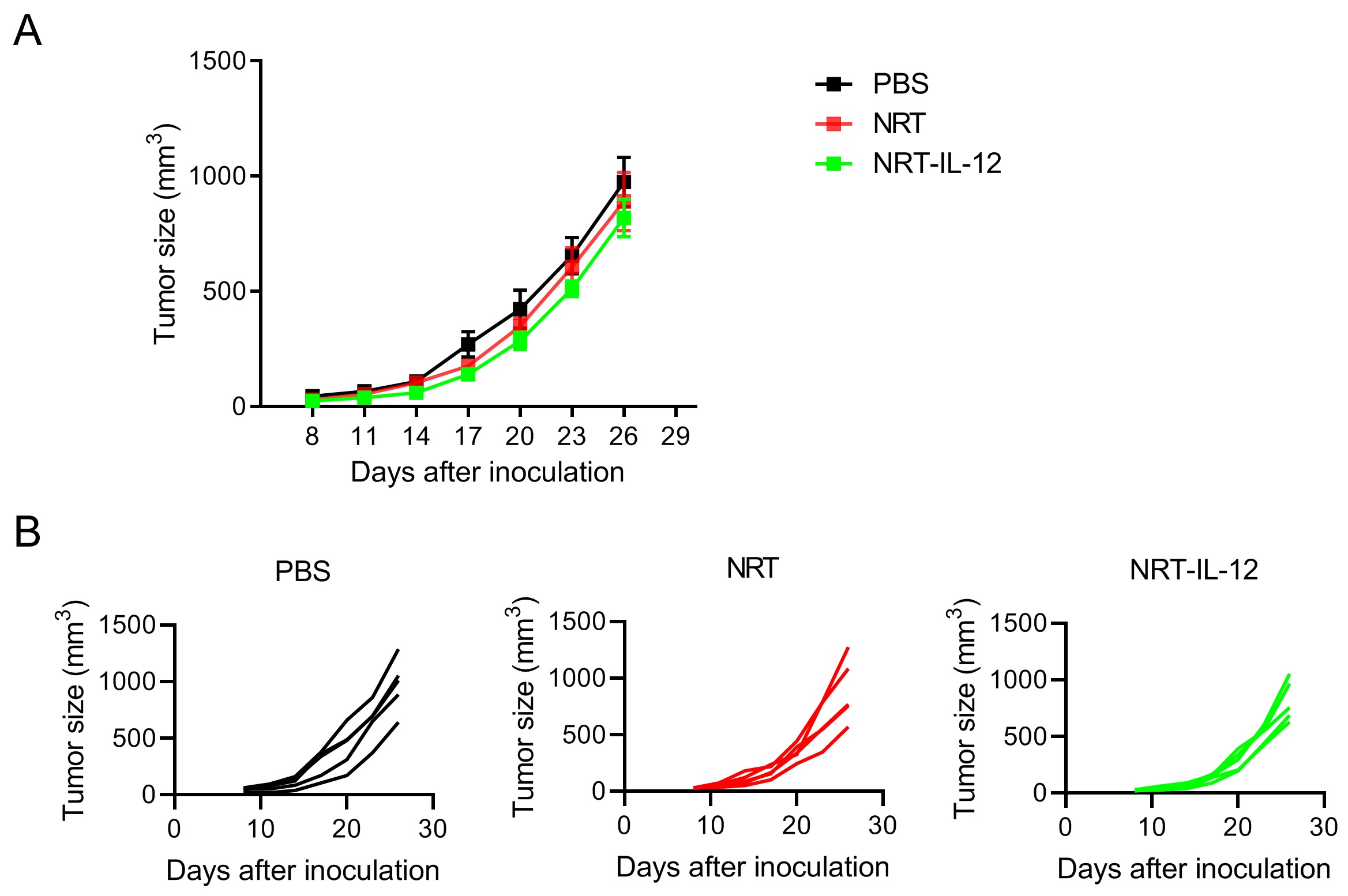

Supplement: ltae010_suppl_Supplementary_Figures_S2 [file ltae010_suppl_supplementary_figures_s2.jpeg]

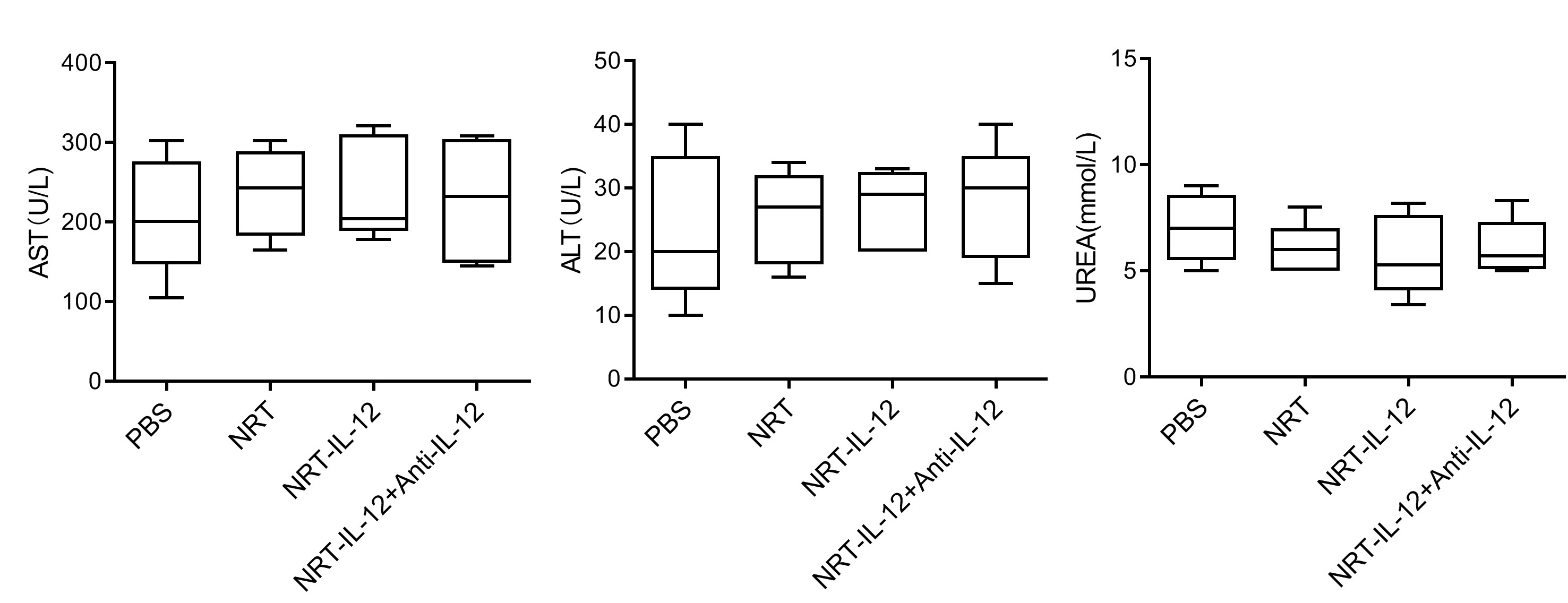

Supplement: ltae010_suppl_Supplementary_Figures_S3 [file ltae010_suppl_supplementary_figures_s3.jpeg]
